# Combining Individual Phenotypes of Feed Intake With Genomic Data to Improve Feed Efficiency in Sea Bass

**DOI:** 10.3389/fgene.2019.00219

**Published:** 2019-03-29

**Authors:** Mathieu Besson, François Allal, Béatrice Chatain, Alain Vergnet, Frédéric Clota, Marc Vandeputte

**Affiliations:** ^1^GABI, INRA, AgroParisTech, Université Paris-Saclay, Jouy-en-Josas, France; ^2^MARBEC, Univ Montpellier, CNRS, Ifremer, IRD, Palavas-les-Flots, France

**Keywords:** aquaculture, feed conversion ratio, fine phenotyping, genomic selection, individual feed intake, restricted feeding, selective breeding

## Abstract

Measuring individual feed intake of fish in farms is complex and precludes selective breeding for feed conversion ratio (FCR). Here, we estimated the individual FCR of 588 sea bass using individual rearing under restricted feeding. These fish were also phenotyped for their weight loss at fasting and muscle fat content that were possibly linked to FCR. The 588 fish were derived from a full factorial mating between parental lines divergently selected for high (F+) or low (F–) weight loss at fasting. The pedigree was known back to the great grand-parents. A subset of 400 offspring and their ancestors were genotyped for 1,110 SNPs which allowed to calculate the genomic heritability of traits. Individual FCR and growth rate in aquarium were both heritable (genomic h^2^ = 0.47 and 0.76, respectively) and strongly genetically correlated (−0.98) meaning that, under restricted feeding, faster growing fish were more efficient. FCR and growth rate in aquariums were also significantly better for fish with both parents from F– (1.38), worse for fish with two parents F+ (1.51) and intermediate for cross breed fish (F+/F– or F–/F+ at 1.46). Muscle fat content was positively genetically correlated to growth rate in aquarium and during fasting. Thus, selecting for higher growth rate in aquarium, lower weight loss during fasting and fatter fish could improve FCR in aquarium. Improving these traits would also improve FCR of fish in normal group rearing conditions, as we showed experimentally that groups composed of fish with good individual FCR were significantly more efficient. The FCR of groups was also better when the fish composing the groups had, on average, lower estimated breeding values for growth rate during fasting (losing less weight). Thus, improving FCR in aquarium and weight loss during fasting is promising to improve FCR of fish in groups but a selection response experiment needs to be done. Finally, we showed that the reliability of estimated breeding values was higher (from+10% up to +125%) with a genomic-based BLUP model than with a traditional pedigree-based BLUP, showing that genomic data would enhance the accuracy of the prediction of EBV of selection candidates.

## Introduction

Improving feed conversion ratio (FCR) is crucial to enhance the sustainability of fish production, as feed is a major economic and environmental cost of fish production (Besson et al., 2017). Improving FCR by selective breeding has already been achieved in terrestrial livestock species by selecting directly on improved growth rate (Knap and Kause, 2018). Faster growing animals are expected to be more efficient as their maintenance cost is proportionally lower than animals growing slowly. In terrestrial livestock, many studies found that the genetic correlation between growth rate and feed intake was negative meaning that improving growth rate generates a correlated decrease of FCR (Knap and Kause, 2018). In fish, however, the genetic correlation between growth rate and FCR is still uncertain. Some studies show no correlation while other show negative correlation (De Verdal et al., 2017; Knap and Kause, 2018). Hence, 64–100% of the genetic variance of FCR is expected not to be explained by growth rate (Knap and Kause, 2018). This large uncertainty on the correlation between growth rate and FCR in fish, and hence on the feasibility of improving FCR through selection for faster growth, is mainly due to the difficulties of measuring individual feed intake.

In livestock, recording individual feed intake was achieved by using electronic feeders (e.g., Gilbert et al., 2007). This kind of device gives access to the feed, located in a closed containment, to a single animal at one time. Then, the device associates the animal to its feed intake using its ear-tag. Using such devices to estimate individual feed intake of fish reared in groups is not possible because of several issues such as the reluctance of fish to enter a closed containment or the difficulty to ensure that the fish eat the entire ration distributed. A first solution to overcome these issues was to estimate feed intake at the scale of a tank composed of fish from the same family. With this method, feed intake is measured at the group level by measuring the amount of feed distributed and by collecting uneaten pellets (e.g., Kolstad et al., 2004). Using separately reared families allows to estimate genetic variability of FCR and then proceed to between-family selection. Family measurements, however, do not enable the estimation of within-family variation, resulting in overestimation of genetic parameters (Doupé and Lymbery, 2003). Estimating within-family variation can be done by measuring FI at the individual fish level by using feed pellets containing radio-opaque glass beads. After a meal, fish are anesthetized and X-rayed, and the pellets in the gastro enteric tract are counted on the radiography (Kause et al., 2006, 2016). This technique is highly accurate to estimate the feed intake of a single meal but it is laborious, as many records on each fish are needed to take into account the variation of feed intake across meals. Consequently, we need to find easier ways to access individual feed intake.

Measuring individual feed intake directly on individual fish over a long period of time would be the best solution to estimate accurate genetic parameters of feed intake or FCR and then develop efficient breeding programs. This was the aim of the present research. To reach this objective, we build an experimental rearing facility of 200 aquariums where fish were reared in isolation and where individual feed intakes were measured accurately over a long period of time. In addition, we chose to feed the fish with a restricted ration because, following the results of studies in rabbit and pigs (Nguyen et al., 2005; Drouilhet et al., 2016), selecting faster growing animal under restricted feeding improved FCR as a correlated response. This is because in a restricted feeding condition, the animals that grow faster are *de facto* the most efficient. The estimation of feed intake in these conditions also enables the calculation of an accurate estimate of individual FCR. Nevertheless, the individual measurement of FCR in aquariums remains laborious and cannot be made on all selection candidates of a fish breeding program, but rather on a (relatively) limited amount of sibs. Thus, we genotyped, with a custom SNP chip, the fish phenotyped for their individual FCR in aquariums to test if genomic information would enhance the estimation of the breeding values of selection candidates. In genomic selection (GS), genotypes and phenotypes of sibs are used in the prediction equations of the GEBVs of the selection candidates that are only genotyped. In aquaculture, several studies have shown the higher performances of GS in terms of genetic gain for traits such as growth (Tsai et al., 2015) or disease resistance (Bangera et al., 2017; Vallejo et al., 2017).

Furthermore, to ensure enough phenotypic variability in the traits measured in aquarium conditions, we used fish divergently selected for their weight loss during fasting to generate our experimental fish population. Weight loss during fasting was shown to be correlated to FCR in rainbow trout (Grima et al., 2008) and to be correlated to residual feed intake (another estimate of feed efficiency) in sea bass (Grima et al., 2010a,b). Weight loss during fasting is, indeed, supposed to be linked to FCR because during fasting, fish use their stored energy to cover maintenance costs. Hence, weight loss during fasting is an indicator of maintenance metabolic rate and selecting for fish with lower weight loss during fasting should, theoretically, reduce FCR due to lower maintenance needs. Furthermore, in the pig industry Knap and Wang (2012) reported positive correlations between back fat depth and FCR, meaning that selection for leaner pigs led to an improvement of FCR. This is because the deposition of fat is less efficient in terms of energy used per unit of wet weight gain than the deposition of protein. Thus, fat content and weight loss at fasting seem promising for the genetic improvement of individual FCR. Consequently, in addition of individual measurement of FCR in aquarium, we also phenotyped the fish for their fat content and their weight loss during fasting in order to identify more traits explaining part of the genetic variation of FCR.

Finally, knowing the individual phenotypes of these fish for FCR, we set up a validation experiment where we tested if the FCR of groups of fish could be explained by their individual performances in aquarium and/or by their weight loss during fasting and their fat content. Here, our objective was (1) to investigate if selection for three indirect traits, weight loss during fasting, fat content, and FCR in aquarium under restricted feeding, could explain the performance of FCR of groups and (2) to test if genomic information could improve the estimation of genetic parameters and breeding values for FCR-related traits.

## Materials and Methods

### Origin of the Fish

*Generation 0*: The animals of G0 were caught from the wild in the West Mediterranean (Gulf of Lions).

*Generation 1*: Forty-one sires and 8 dams randomly chosen from G0 were mated in a full factorial mating design to create the G1 generation (Grima et al., 2010b). The G1 individuals (1,912 fish) were phenotyped for their growth performance during two fasting periods of 3 weeks following normal feeding periods of 3 weeks. The trait measured was the average thermal growth coefficient (TGC) from the two periods, corrected for the effects of initial weight and initial TGC (FDcorr) (Grima et al., 2010b).

*Generation 2*: Broodstock fish were selected from the 1,912 candidates of G1 based on their phenotypes for FDcorr (mass selection) to create generation G2. Twenty sires and 5 dams with the lowest FDcorr (losing much weight at fasting) were mated in a full factorial mating design to create the F+ line. In parallel, 20 sires, and 5 dams with the highest FDcorr (loosing less weight during fasting) were mated in a full factorial mating design to create the F– line (Daulé et al., 2014). The average selection differential was +1.49 phenotypic standard deviations (σ_P_) in FDcorr for the F– dams, +2.25 σ_P_ for F– sires, −1.81 σ_P_ for the F+ dams and −1.74 σ_P_ for the F+ sires. A total of 1,037 individuals of G2 generated from these matings were phenotyped for FDcorr during three feed deprivation periods of 3 weeks.

*Generation 3*: Two G1 dams (one from the F+ line and one from the F– line) were mated with 30 G2 sires (15 from F+ 15 sires from F–) in a full factorial mating design. We had to pick females from G1 because there were no females from G2 ready to spawn at the time of the mating. Both sire and dams were chosen based on their FDcorr phenotypes. The selection differential was −1.70 σ_p_ for the F+ dam and +1.20 σ_p_ for the F– dam while the selection differential was −1.82 σ_p_ for the F+ sires and +1.49 σ_p_ for the F– sires.

### Initial Growing Period

After artificial fertilization, all G3 families were mixed and kept in 2 replicate tanks at 48 h post-fertilization. At 100 days post-fertilization (dpf), fish were transferred to two 1.5 m^3^ fiberglass tanks. At 185 dpf (mean weight = 13.1 g), 660 fish from one tank were individually tagged with the passive integrated transponder. At 276 dpf (mean weight = 33.22 g), 350 fish from the second tank were individually tagged and mixed with the previously tagged fish. At tagging (in total 1,010 G3 fish tagged), a piece of fin from each fish was collected for DNA extraction for parentage assignment and genotyping.

### Phenotyping

**Figure 1:**
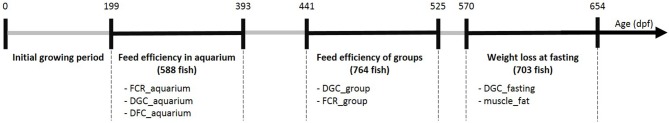
Summary of the experiments realized on G3 fish at different ages (days post-fertilization). For each experimental period we listed the phenotypes available and the number of individuals phenotyped (between brackets). FCR refers to feed conversion ratio, DGC to daily growth coefficient, DFC to daily feed intake coefficient and muscle_fat refers to muscle fat content.

The G3 fish went through several phenotyping experiments that are described below and summarized in Figure 1.

#### Individual Feed Efficiency in Aquarium Under Restricted Feeding

**Figure 2:**
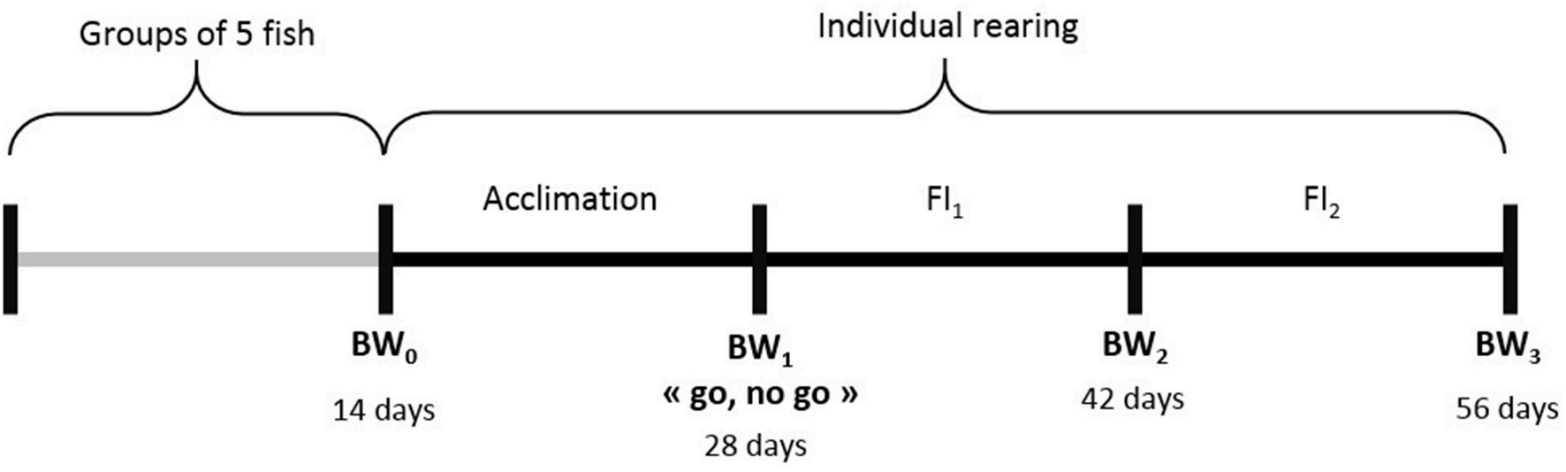
Experimental scheme for an individual fish in aquariums. BW_i_ = successive measurements of individual body weight, FI_i_ = cumulated feed intake over a given period of isolation.

Two hundred 10 l aquariums were used, in a recirculation system where natural salinity sea water was kept at 21°C. Individuals from G3 were first reared in groups of five fish in each aquarium, to enable adaptation of the fish to their new environment. After 14 days, they were weighted and randomly split into individual aquariums. After 14 days of acclimation in isolation, the fish were weighted again in a “go, no go” step. The fish that lost weight during this period were removed. The remaining ones were kept in aquariums for 2 more periods of 14 days. In total, a “successful” fish stayed 56 days in aquarium and was weighted 4 times (Figure 2) before being replaced by another fish in the aquarium. For the first batch, the age at starting of the fish was 199 dpf while, for the last batch, fish started at 324 dpf. Individual BW at each measurement was used to estimate the individual feeding ration for the following period. This ration (1.3% BW/day) was half the standard ration (2.6% BW/day) given by the feed manufacturer (see feed composition in Supplementary Table 1). We chose a high level of restriction because, in aquarium conditions, fish do not express their full feed intake potential. Some pre-tests showed that their *ad libitum* in aquariums was lower than in normal rearing conditions. Fish were fed automatically once a day in the morning (9.00 a.m.) with the whole of this daily ration. Every afternoon, uneaten pellets were counted in each aquarium and then removed. The number of pellets was then converted to grams (1 pellet ≈ 0.00925 g). Among the 831 G3 fish tested in aquariums, 185 fish did not pass the “go, no go” step. Thus, 646 fish were evaluated for individual feed intake over 2 periods of 14 days. For those 646 fish, we calculated their cumulated FCR (noted as FCR_aquarium) using their cumulated weight gain (BWG = BW_3_-BW_1_) and cumulated feed intake (cumFI = FI_1_+FI_2_). We excluded fish with aberrant performances: 6 fish with negative FCR_aquarium and 52 fish with FCR_aquarium higher than 2.60. Applying these thresholds, we could keep 588 fish with data available for FCR_aquarium, DGC_aquarium and DFC_aquarium, calculated as follows.

(1) $$\begin{array}{l} \text{FCR\_aquarium}=\frac{\text{BWG}}{\text{cumFI}} \end{array}$$

(2) $$\begin{array}{l} \text{DGC\_aquarium}=\frac{{\text{BW}}_{3}^{\frac{1}{3}}-{\text{BW}}_{1}^{\frac{1}{3}}}{28}\;\times 100 \end{array}$$

(3) $$\begin{array}{l} \text{DFC\_aquarium}=\frac{{\text{cumFI}}^{\frac{1}{3}}-{\text{BW}}_{1}^{\frac{1}{3}}}{28}\;\times 100 \end{array}$$

Where DGC is the daily growth coefficient, DFC, is the daily feed intake coefficient calculated following Janssen et al. (2017), BWG is the weight gain during the 2 isolation periods in aquarium and 28 is the duration in days between the measurements of BW_1_ and BW_3_. All traits were log transformed to enhance homogeneity of variance and to linearize the relationships between the traits.

#### Weight Loss at Fasting

At 570 dpf (177 days after the last fish ended phenotyping in aquarium), 764 fish previously tested in aquariums, were phenotyped for their tolerance to fasting in a 5 m^3^ fiberglass tank. The tolerance to fasting was calculated as the average (negative) daily growth coefficient over two fasting periods of 3 weeks (DGC_fasting). These two fasting periods (fasting_P1 and fasting_P2) were separated by a period of 3 weeks of refeeding, similar to Grima et al. (2010b), where fish were fed *ad libitum* using a self-feeder with a standard commercial diet.

#### Feed Efficiency of Groups

To test the link between individual feed efficiency and group feed efficiency, the 588 fish phenotyped in aquariums were split in groups according to their individual performances in aquariums as follow:

- First, 6 groups of 98 fish were constituted based on categories of relative feed intake (cumFI/BW_1_).
- Second, in each group, the 98 individuals were split in 2 sub-groups of 49 individuals based on their relative weight gain (BW_3_-BW_1_)/BW_1_.

**Figure 3:**
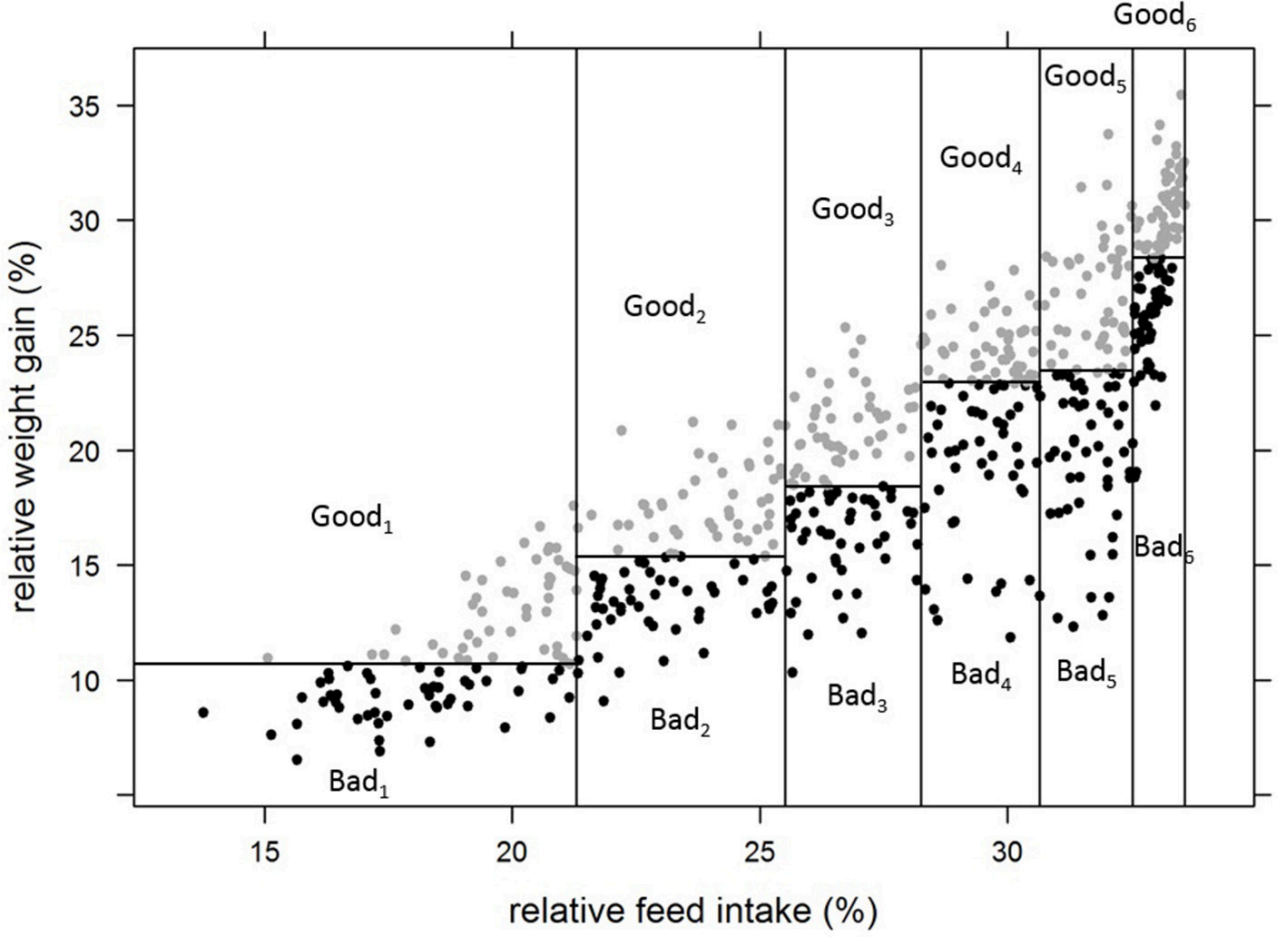
Formation of groups of fish for group testing of FCR, based on their individual performance for relative feed intake and relative weight gain in aquariums. Good_i_ refers to the sub-groups of fish with better (lower) FCR_aquarium for a given group of relative feed intake. Bad_i_ refers to the sub-groups of fish with worse (higher) FCR_aquarium for the same group of relative feed intake.

Thus, 12 groups of individually tested fish were constituted, with one “high FCR” and one “low FCR” sub-group for each of the 6 categories of relative feed intake (Figure 3). In addition, we formed four more groups of 44 fish with the fish that lost weight during the first period of rearing in isolation in aquariums. These four groups were made to test if the non-acclimation to individual rearing could be linked to group FCR. In total, 764 fish were stocked in 16 tanks of 2 m^2^ covered by opaque plastic curtains to avoid disturbance. Fish were fed once a day *ad libitum* using an automatic feeder delivering the daily ration in 20 portions over 6 h and 20 min. The frequency of distribution was every 5 min for the 5 first portions, every 10 min for the next 5 portions, then every 20 min for the next 5 portions and finally, every 30 min for the final 5 portions. The feeders were filled with a known amount of pellets. Uneaten pellets were collected in the fecal trap of each tank. Every day, at the end of the feeding period, each fecal traps were checked. If pellets were found, it meant that fish of the tank reached *ad libitum*. If a fecal trap was empty or only few pellets were present, an additional portion was given to the tank by activating the feeder manually. Additional portions were then given every 30 min until pellets were present in the fecal trap meaning that *ad libitum* was reached. Uneaten pellets of all tanks were then collected, photographed and counted using ImageJ (Abràmoff et al., 2004). The group FCR experiment lasted for 4 periods of 3 weeks from 441 to 525 dpf (48 days after the last fish ended phenotyping in aquarium); the first 3 weeks were considered an acclimation period followed by three testing periods (group_P1, group_P2, group_P3) where daily group feed intake was recorded. Feed intake was therefore also available for a period a 9 weeks (group_full). At the beginning of the first period and at the end of each of the three testing periods, all fish were weighted in order to estimate the weight gain of the groups. Finally, using the daily feed intake of the groups and the body weight gain of the groups over the periods of 3 weeks, the average FCR of each group (FCR_group) was estimated for each period.

#### Fat Content

We measured the dorsal muscle fat content of the fish using indirect ultrasonic measurement (Distell Fish Fatmeter, FM 692) according to the method described by Douirin et al. (1998). Briefly, after a fish had been anesthetized and weighted, the Fatmeter was applied on each side of the fish. We only measured once on each side of the fish because they were not big enough to permit several measurements on each side. Thus, fat measurements were the average of two measurements. Four fat measurements took place during the fasting experiment, after each period of 3 weeks at 591, 612, 633, and 654 dpf. Thus, from the fasting experiment, we calculated the average fat content after feeding periods (muscle_fat). Muscle_fat measurements were log transformed to reduce heteroscedasticity.

### Genetic Analysis

#### Genotyping and Parentage Assignment

The 50 grand-parents from G1 and the 49 great grandparents from G0 were genotyped with an iSelect Custom Infinium Illumina® European sea bass array of 2.722 SNP (Faggion et al., 2018). Then, with a similar array of 3,987 SNP, we genotyped:

- 400 fish out of the 588 with valid phenotypes in aquarium. The 400 are those with the highest relative feed intake.
- 66 fish that lost weight after the first 2 weeks alone in aquarium and which also took part in the experiment of feed efficiency of groups.
- And their 32 parents (2 G1 females and 30 G2 males).

This second array uses the same markers as the original 2,722 SNP array plus 1,265 duplicated markers that were ineffective on the original array due to bioinformatics problems that occurred during probe design. Once the animals were genotyped, the first step to create the SNP dataset used in our genomic analysis was to apply classic quality control ignoring all SNPs with a MAF inferior to 5% and a call rate inferior to 90% in the G3 animals. This quality control resulted in keeping 2,100 SNP for G3 individuals and their parents. From this 2,100, we discarded the original version of all duplicated markers, which resulted in keeping 1,923 SNP markers for G3 individuals and their parents. Then, we kept only the markers that were in common between both chips, representing 1,110 SNP. Finally, we discarded all animals for which the call rate (number of SNP genotyped over the number of SNP on the array) was lower than 90% indicating potential quality issue of the DNA sample. This resulted in keeping 5 individuals of G0 (out of 49), 52 individuals of G1 (out of 52), 30 fish of G2 (out of 30), and 399 fish of G3 (out of 400).

The pedigree of G1 and G2 fish was previously retrieved by Grima et al. (2010a) and Daulé et al. (2014) using microsatellites markers and VITASSIGN, an exclusion-based parentage assignment software (Vandeputte et al., 2006). We also used VITASSIGN to retrieve the pedigree of all the 764 fish of G3 that were tested in the group experiment. Among those fish, 463 fish (out of 466 genotyped on the SNP array) were correctly assigned considering a percentage of mismatches lower than 2% (99.3% success). Then, among the remaining 298 fish typed for 12 microsatellites markers (188 fish with valid phenotypes in aquariums and 110 not phenotyped in aquarium), 286 fish could be assigned (96% success) to a single parental pairs.

#### Breeding Value Estimation

Variance components and estimated breeding values for all traits were computed based on multivariate linear mixed animal models. In these multivariate models, we always included DGC_fasting as a “reference” trait because DGC_fasting was measured on all G1, G2, and G3 fish, even those not selected to create the next generation(s). This allowed to integrate the selection process realized on weight loss at fasting in the estimation of variance components. Thus, 1278 fish of G1, 1029 fish of G2, and 701 fish of G3, all with DGC_fasting phenotypes and pedigree, were included in all models. For DGC_fasting, the linear model included the fixed effect of the generation as DGC_fasting was measured at different ages in different conditions across generations. Then, in the multivariate models, the other traits included were the traits only measured on G3 fish (e.g., DGC_aquarium, or fat_fasting). The models were fitted by restricted maximum likelihood in AIREMLF90 (Misztal et al., 2002) to compute the classical heritability's using pedigree and the genomic heritability's using genomic information. The breeding values were also computed with classical pedigree-based BLUP and single-step GBLUP (ssGBLUP) using the genomic relationship matrix. The conventional pedigree-based EBVs were estimated using the following model:

$$\begin{array}{l} y=Xb+Zu+e \end{array}$$

Where **y** is the vector of phenotypes, **b** is the vector of fixed effects (batch, rack, line, and column for the phenotypes measured in aquariums) and **X** an appropriate incidence matrix, **u** is the vector of random additive genetic animal effects, **Z** the appropriate incidence matrix and **e** is vector of random error variance. The additive (animal) genetic effects were assumed to follow *N*(**0**, *V* ⊗ *A***)**, with **V** the genetic (co) variance matrix between traits and **A** the numerator relationship matrix relating all animals in the pedigree, while the residual effects were assumed to follow *N*(**0**, *R*⊗*I***)**, **R** the residual (co) variance matrix between traits and **I** an appropriate identity matrix. The SNP based EBV (GEBV) was estimated using a single-step GBLUP (ssGBLUP) combining pedigree, genomic and phenotypic information (Legarra et al., 2014). In ssGBLUP, the relations between non-genotyped fish are based on the numerator relationship matrix (**A** matrix) derived from the pedigree, while the relations between fish with genotypes are based on the genomic relationship matrix described by VanRaden (2008) (**G** matrix). Apart from that, the general model (*y* = *Xb*+*Zu*+*e*) remains the same as in PBLUP.

#### Cross Validation Scheme to Test Predictive Abilities

The predictive abilities of the different models described above (PBLUP and ssGBLUP), depending on the number of fish phenotyped were assessed using a cross validation scheme. The model tested was a bivariate model including the phenotypes of 3,008 fish over 3 generations for DGC_fasting and the phenotypes of 588 fish of G3 for log(DGC_aquarium), We tested log(DGC_aquarium) as this trait was considered as an adequate variable describing feed efficiency (see Results). We included the generation as fixed effect for DGC_fasting while we used the batch, the rack and the column as fixed effects for log(DGC_aquarium). The cross validation procedure followed three steps:

- The fish phenotyped and genotyped (400 fish) were randomly split into two groups; a given number of fish was set as the training group and the remaining fish were set as validation group.
- Then, the EBV and GEBV for log(DGC_aquarium) of all of the 400 fish were predicted while masking the phenotype of the validation group (phenotype set missing). In order to test the predictive ability of our models depending on the number of fish phenotyped and genotyped, we tested a decreasing number of individuals in the training group, from 360 to 280, 200, 120, and 40.
- Then, for each number of fish in training group, we calculated the squared correlation between the EBV or the GEBV and the phenotypes corrected for fixed effects ($r_{EBV,y}^{2}$) of the fish in the validation group. We also calculated the Spearman rank correlation to estimate the degree of similarity between the rankings of fish based their phenotypes or their EBV or GEBV.

In addition, to reduce to stochastic effects we replicated the cross validation scheme 300 times for each number of individuals in training and validation group. Thus, we could calculated the average $r_{EBV,y}^{2}$ and average Spearman rank correlation over the 300 repetitions for each of the five sizes of training population (40, 120, 120, 280, and 360 fish). The average $r_{EBV,y}^{2}$ was used to estimate the reliability (*R*_*EBV, BV*_) of PBLUP and ssGBLUP models using the following formula as in Bangera et al. (2017):

(4) $$\begin{array}{l} R_{EBV,BV}=\frac{\bar{r_{EBV,y}^{2}}}{h^{2}} \end{array}$$

Where, $\bar{r_{EBV,y}^{2}}$ is the average over 300 repetition of the squared correlation between the predicted EBV and GEBV for log(DGC_aquarium) for all the fish in the validation group and the recorded DGC_aquarium, corrected for fixed effects, and h^2^ is the heritability of log(DGC_aquarium) estimated using pedigree including all fish with phenotypes (h^2^ = 0.39).

### Statistical Analysis

In this section, all complete models are described. All linear models were analyzed using the lm procedure of R (R Development Core Team, 2008).

#### Individual Performances in Aquarium

The individual performances in aquarium were studied using the following linear model:

$$\begin{array}{l} {\text{y}}_{\text{ijklmnop}}=\mu +{\text{batch}}_{\text{j}}+{\text{rack}}_{\text{k}}+\text{lin}{\text{e}}_{\text{l}}+{\text{column}}_{\text{m}}\\ \;+{\text{sire\_origin}}_{\text{n}}+{\text{dam\_origin}}_{\text{o}}+{\text{sire\_dam\_origin}}_{\text{no}}\\ \;+{\text{e}}_{\text{ijklmnop}} \end{array}$$

Where, y_jklmnop_ is the (log transformed) trait of interest (FCR_aquarium, DGC_aquarium or DFC_aquarium), μ is the overall mean, batch_j_ is the fixed effect of the batch in which the fish has been phenotyped in aquariums (1–10). Rack_k_, line_l_, and Column_m_ are the fixed effect of the physical position of the aquarium in which the fish have been phenotyped. There were 4 racks of 50 tanks in 5 lines and 10 columns. sire_origin_n_ and dam_origin_o_ are the fixed effect of the line of origin of sires and dams with 2 levels each (F+ and F–). sire_dam_origin_no_ is the interaction between sire and dam origins. Finally, e_ijklmnop_ is the random residual effect. From this complete model, we used the boot.stepAIC function in R to find out which fixed effects had to be included in the model for the genomic analysis for the different phenotypes observed. The boot.stepAIC function looks for the model with the lowest Akaike information criterion (AIC) (Austin and Tu, 2004).

#### Weight Loss at Fasting

First, the effect of parental origin and fat content on weight loss at fasting of G3 fish was analyzed using the following linear model:

$$\begin{array}{l} {\text{DGC\_fasting}}_{\text{ijk}}=\mu +\beta \;{\text{muscle\_fat}}_{\text{k}}+{\text{sire\_origin}}_{\text{i}}\\ \;+{\text{dam\_origin}}_{\text{j}}+{\text{sire\_dam\_origin}}_{\text{ij}}+\;{\text{e}}_{\text{ijk}} \end{array}$$

Where μ is the overall mean, muscle_fat_k_ is the covariate describing the effect of muscle fat content measured before fasting on individual k, sire_origin_i_ is the fixed effect of sire origin (F+ or F–), dam_origin_j_ is the fixed effect of dam origin (F+ or F–), sire_dam_origin_ij_ is the interaction between sire and dam origins and e_ijk_ is the random residual effect.

Second, we tested if the selection process over 2.5 generations for lower or higher weight loss during fasting was efficient by comparing the genomic estimated breeding value (GEBV) of fish from the different parental origins, F+/F+ (both parents of F+ line), F+/F– and F–/F+ (one parent of F+ and one parent from F–) and F–/F– (both parents of F– line) within G2 or G3 fish using the following models:

$$\begin{array}{l} {\text{GEBV\_DGC\_fasting}}_{\text{ij}}=\mu +{\text{line\_origin}}_{\text{i}}+\;{\text{e}}_{\text{ij}} \end{array}$$

Where μ is the overall mean, line_origin_j_ is the effect of the line of origin of the fish j with two levels (F+/F+ and F–/F–) for G2 fish and with four levels in G3 (F+/F+, F–/F+, F+/F–, and F–/F–). e_ij_ is the random residual effect. GEBVs of DGC_fasting of G2 and G3 fish were calculated using single-step GBLUP procedure.

#### Feed Efficiency of Groups

The aim of the experiment in groups was to test whether individual performances measured in aquarium and during fasting could predict the performance of fish in groups. As a first analysis, therefore, we applied a paired sample *t*-test to compare FCR_group between tanks of fish with higher or lower relative weight gain within each groups of relative feed intake (6 groups). Then, we tested if the average phenotypic performances in aquarium of the fish composing a tank could predict the FCR of the tank (FCR_group). To do so, we computed the group mean performance to obtain avg_DGC_aquarium, avg_DFC_aquarium and, for each periods of 3 weeks (group_P1, group_P2, and group_P3) and for the combined period of 9 weeks (group_full), we tested the following model:

$$\begin{array}{l} {\text{FCR\_group}}_{\text{i}}=\mu +\beta 1\;avg\text{\_}DGC\text{\_}aquarium_{\text{i}}\\ \;+\beta 2\;{\text{avg\_DFC\_aquarium}}_{\text{i }+}{\text{e}}_{\text{i}} \end{array}$$

Where μ is the overall mean, avg_DGC_aquarium_i_ and avg_DFC_aquarium_i_, are the average over tank i of the DGC_aquarium and DFC_aquarium, of every fish in tank i and e_i_ is the random residual effect. For this analysis we could test the FCR_group of 12 tanks out of 16 because 4 tanks were composed of fish that were not phenotyped in aquariums.

Then, we tested if the average (GEBV which are described in the Genetic Analysis section) of DGC_fasting of the fish composing a tank could predict the FCR of the tank (FCR_group). To do so, we computed the group mean GEBV to obtain avg_GEBV_DGC_fasting for each periods of 3 weeks (group_P1, group_P2, and group_P3) and for the combined period of 9 weeks (group_full), we tested the following model:

$$\begin{array}{l} {\text{FCR\_group}}_{\text{i}}=\mu +\beta 1\;{\text{avg\_GEBV\_DGC\_fasting}}_{\text{i}}+{\text{e}}_{\text{i}} \end{array}$$

For this analysis we could test the FCR_group of the 16 tanks for group_P1 and group_P2 and 14 tanks for group_P3 and group_full. We could get data only for 14 tanks during group_P3 because feed wasted could not be collected for two tanks for a day due to operating mistake.

## Results

### Feed Efficiency in Aquariums

**Figure 4:**
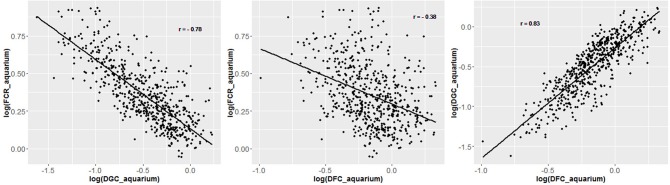
Regression plot of individual traits measured in the aquarium experiment and corrected for fixed effects. Data are log transformed to enhance linearity of regression.

**Figure 5:**
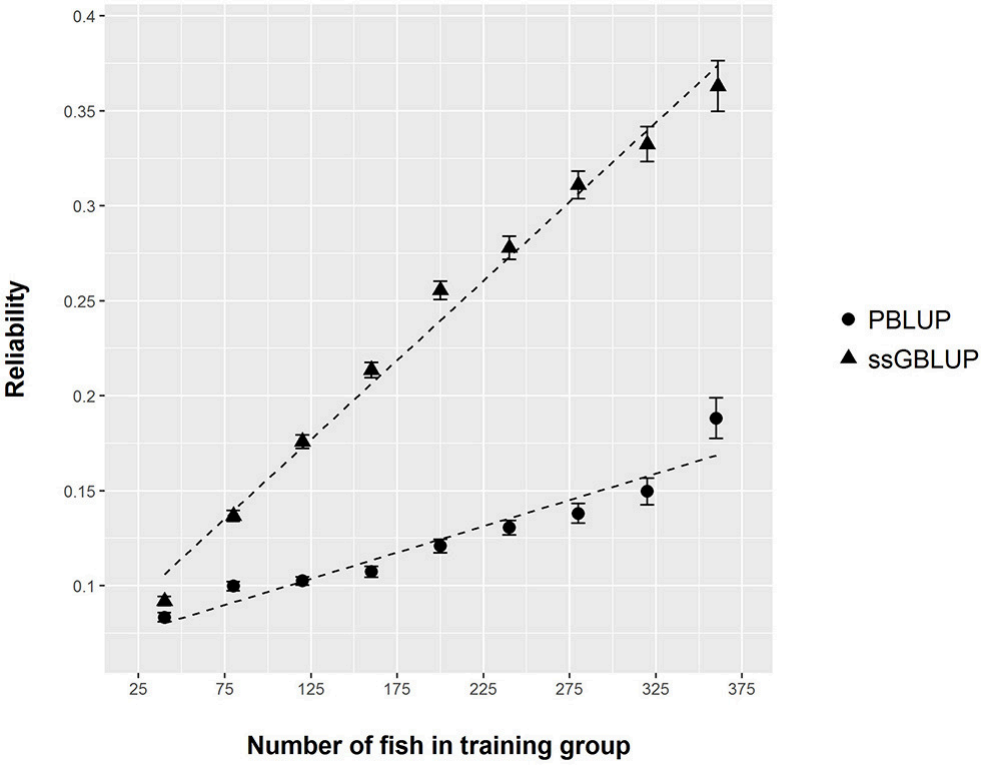
Reliability of PBLUP and ssGBLUP models for the prediction of log(DGC_aquarium), estimated by 300 repetitions of cross validation, as a function of the number of fish in the training group.

From the phenotyping experiment in aquarium, we could measure significant phenotypic variances for FCR_aquarium, DGC_aquarium and DFC_aquarium (Table 1). There was a significant effect of sire origin on the three traits, log(FCR_aquarium) [*F*_(1, 553)_ = 11.64, *P* <0.0001], log(DGC_aquarium) [*F*_(1, 553)_ = 13.97, *p* <0.0001] and log(DFC_aquarium) [*F*_(1, 553)_ = 8.31, *p* =.0042]. The effect of the origin of the dam was also significant for log(FCR_aquarium) [*F*_(1, 553)_ = 4.86, *p* =^.^028]. The interaction effect between sire and dam origin was not significant for any trait (Table 2). This means that the fish with two F- parents were more efficient, were growing faster and were eating more than fish with only one F– parent (dam or sire), or with two F+ parents. In addition, the three traits measured in aquariums displayed large phenotypic correlation [*r* = −0.78 between log(FCR_aquarium) and log(DGC_aquarium); *r* = 0.83 between log(DGC_aquarium) and log(DFC_aquarium) and moderate phenotypic correlation between log(FCR_aquarium) and log(DFC_aquarium)] (*r* = −0.38, Figure 4). These results show that, in aquariums under restricted feeding conditions, the fish that grow faster have a better (lower) FCR. The three main traits measured in aquariums were heritable, and the heritability estimate was greater when using genomic data than using pedigree only (Tables 3, 4). Furthermore, the three traits were all strongly genetically correlated, both with pedigree or genomic data but again the genetic correlations were greater with genomic data (Tables 3, 4). The reliability of the ssGBLUP model to predict the GEBV of DGC_aquarium increased with an increasing number of animal used as training (Figure 5). Both the intercept and the slope of the regression of predictive ability on training population size were higher with ssGBLUP than with PBLUP, meaning that ssGBLUP was always more predictive than PBLUP and that including more individuals in the training group increased the predictive ability of ssGBLUP more than the predictive ability of PBLUP (Figure 5). Furthermore, in PBLUP models, the Spearman rank correlation was constant from 0.19 (0.005) when 40 fish were in the training group to 0.20 (0.008) when 360 were in the training group whereas the Spearman rank correlation increased in ssGBLUP from 0.20 (0.002) to 0.36 (0.007).

**Table 1:** Overview of phenotypic results in aquariums.

	**Mean**	**Median**	**CV%**
FCR_aquarium	1.38	1.46	21.9
DGC_aquarium	0.65	0.63	32.9
DFC_aquarium	0.87	0.85	21.2

**Table 2:** Least square means (±s.e.) of individual performance of G3 fish in aquariums [log(FCR_aquarium), log(DGC_aquarium), and log(DFC_aquarium)] as function of the line of origin of sires and dams.

	**Sire origin**		**Dam origin**		**Significance levels**						
	**F+**	**F–**	**F+**	**F–**	**Batch**	**Rack**	**Column**	**Line**	**Sire_origin**	**Dam_origin**	**Sire_origin × Dam_origin**
Log(FCR_aquarium)	0.40 ± 0.014	0.35 ± 0.013	0.39 ± 0.013	0.36 ± 0.014	<0.0001	0.02	0.001	0.29	0.0007	0.028	0.29
Log(DGC_aquarium)	−0.54 ± 0.023	−0.43 ± 0.022	−0.49 ± 0.022	−0.49 ± 0.024	<0.0001	0.0005	0.0003	0.28	0.0002	0.90	0.60
Log(DFC_aquarium)	−0.18 ± 0.014	−0.13 ± 0.014	0.14 ± 0.013	0.17 ± 0.014	<0.0001	0.0003	0.001	0.13	0.004	0.13	0.90

**Table 3:** Genetic parameters of traits measured in aquariums calculated using pedigree information.

	**log (FCR_aquarium)**	**log (DGC_aquarium)**	**log (DFC_aquarium)**
log(FCR_aquarium)	0.25 ± 0.1	−0.95 ± 0.26	−0.83 ± 0.47
log(DGC_aquarium)		0.39 ± 0.13	0.96 ± 0.36
log(DFC_aquarium)			0.42 ± 0.14

*Heritability (±s.e.) on the diagonal and genetic correlations (±s.e.) above the diagonal*.

**Table 4:** Genetic parameters of traits measured in aquariums calculated using genomic data.

	**log (FCR_aquarium)**	**log (DGC_aquarium)**	**log (DFC_aquarium)**
log(FCR_aquarium)	0.47 ± 0.07	−0.98 ± 0.04	−0.90 ± 0.07
log(DGC_aquarium)		0.75 ± 0.05	0.95 ± 0.02
log(DFC_aquarium)			0.57 ± 0.07

*Heritability (±s.e.) are in diagonal and the genetic correlations are above the diagonal*.

### Weight Loss at Fasting

**Figure 6:**
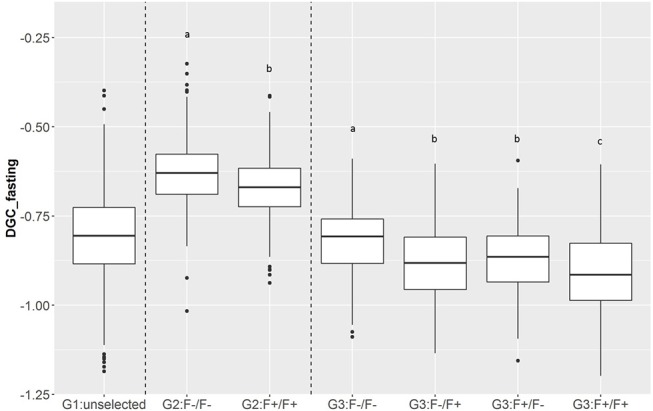
Boxplot of DGC measured during fasting periods (DGC_fasting) as function of generations (G1, G2, and G3) and parental origins. The letters shows the significant differences between parental origins within generations.

**Figure 7:**
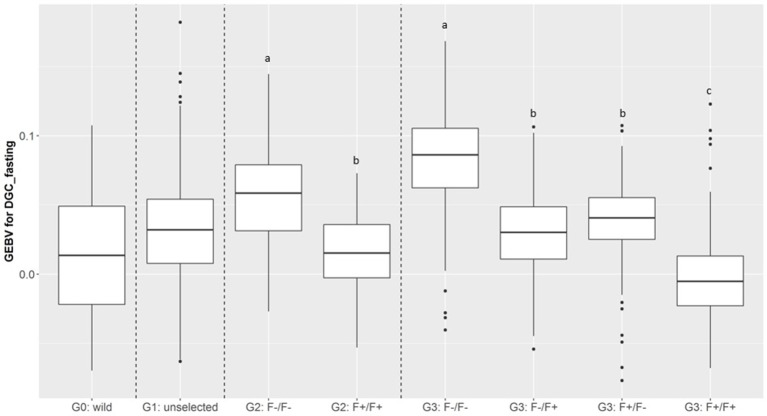
Boxplot of genomic estimated breeding value (GEBV) for DGC_fasting as a function of generation and parental origins. The letters shows the significant differences between parental origins within generations.

In total, 701 of G3 fish were phenotyped for their tolerance of fasting calculated as the average (negative) daily growth coefficient over two consecutive feed deprivation periods (DGC_fasting). Figure 6 is a boxplot of DGC_fasting for each generation as function of parental origin. In G2 fish, we observed a significant divergence in phenotypes. This significant divergence in DGC_fasting was also observed in the next generation G3 between all parental origins. The differences in the average DGC_fasting between generations is explained by the fact that the experiments were done separately and at different ages for the different generations. Furthermore, within G3 fish we showed that DGC_fasting was significantly affected by the fat content before fasting periods, muscle_fat [*F*_(1, 693)_ = 6.10, *p* = 0.013], the dam origin, F+ or F– [*F*_(1, 693)_ = 13.38, *p* = 0.0002], and the interaction between dam origin and muscle_fat [*F*_(1, 693)_ = 6.02, *p* = 0.014] (more information in Supplementary Table 2). The genomic heritability of DGC_fasting was moderate and the heritability of log(muscle_fat) was high (Table 5). Also, log(muscle_fat) was positively genetically correlated with DGC_fasting (0.34 ± 0.12). In addition, the GEBVs were significantly different between parental origins in G2 [*F*_(1, 1, 027)_ = 497.9, *p* < 0.0001 for G2] and G3 [*F*_(3, 747)_ = 238.1, *p* < 0.0001 for G3 fish] (Figure 7). In G2, fish from the F–/F– parents had higher GEBV for DGC_fasting than fish with F+/F+ parents. This trend was confirmed in G3 fish, where there were differences in GEBV of DGC_fasting between the divergent lines F–/F+ and F+/F+, between F–/F– and hybrid lines (F–/F+ andF+/F–), between F+/F+ and hybrid lines (F–/F+ and F+/F–) but there were no differences between hybrid lines F+/F– and F–/F+ (Tukey LSD test, Supplementary Table 3).

**Table 5:** Genetic parameters of traits measured during feed deprivation periods [DGC_fasting and log(muscle_fat)] calculated using genomic data.

	**DGC_fasting**	**log(muscle_fat)**
DGC_fasting	0.21 ± 0.03	0.34 ± 0.12
log(fat_fasting)		0.75 ± 0.04

*The heritabilities (±s.e.) are in diagonal and the genetic correlations (±s.e.) are above the diagonal*.

### Feed Efficiency of Groups

**Figure 8:**
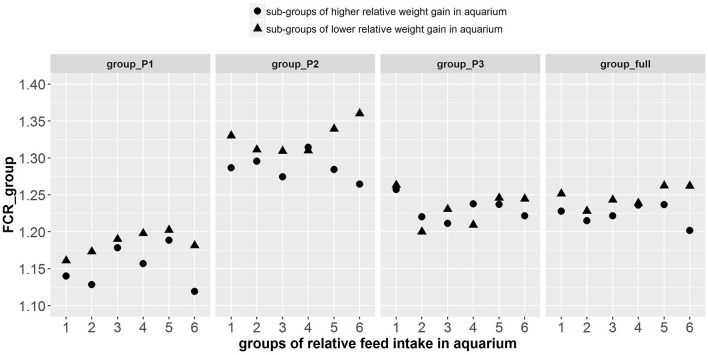
Group FCR results of the 12 tanks composed of the fish phenotyped individually in aquarium. On the abscissa are the 6 different groups of fish made based on the relative feed intake of individual fish in aquariums. The different shapes of points among the 6 groups represent the sub-groups of fish with higher (▴) and lower (•) relative weight gain in aquarium. Group FCR results are given for 3 consecutive periods of 3 weeks as well as for the overall period of 9 weeks.

The paired sample *t*-test showed that there were significant differences in FCR_group between tanks composed of fish with higher relative weight gain (thus lower individual FCR) and tanks composed of fish with lower relative weight gain (thus higher individual FCR) during periods group_P1 [*t*_(1, 5)_ = −3.94, *p* < 0.005], group_P2 [*t*_(1, 5)_ = −2.35, *p* < 0.033] and for the combined group_full [*t*_(1, 5)_ = −3.1, *p* < 0.014]. However, in group_P3, differences in the FCR_group were not significant (Figure 8). Aadditionally, there was a significant effect of log(avg_DGC_aquarium) and log(avg_DFC_aquarium) on FCR_groups during group_P1, group_P2, and group_full (Supplementary Table 4). More particularly, the tanks composed of fish with higher log(DGC_aquarium) and lower log(DFC_aquarium) were more efficient in these periods. Finally, there was a significant effect of avg_GEBV_DGC_fasting on FCR_groups during group_P1, group_P2, and group_full, (Supplementary Table 5). The tanks composed of fish with lower GEBV of weight loss during fasting were more efficient (lower FCR) in these periods.

### Genomic Correlations Between Growth Rates and Fat Content Across Experiments

The genetic correlations between DGC_aquarium and the growths rates measured in the fasting and in the group experiments were not significantly different than zero (Table 6). At the contrary, there were moderate negative genetic correlations between DGC_fasting and DGC_group (Table 6). Additionally, there were moderate to high positive genetic correlations between the different DGC and fat measured before fasting periods, which had a high heritability. More particularly, the genetic correlation was 0.39 between log(DGC_aquarium) and log(muscle_fat) (Table 6).

**Table 6:** Genetic parameters of log(muscle_fat) and daily growth coefficient (DGC) measured during different experiment, in aquarium (DGC_aquarium), during fasting experiment (DGC_fasting), and during the experiment in groups (DGC_group).

	**Log (DGC_aquarium)**	**DGC_fasting**	**DGC_group**	**Log (muscle_fasting)**
Log(DGC_aquarium)	**0.75 ± 0.05**	−0.13 ± 0.16	0.13 ± 0.10	**0.39 ± 0.09**
DGC_fasting		**0.21 ± 0.03**	−0.20 ± 0.13	**0.29 ± 0.13**
DGC_group			**0.70 ± 0.03**	**0.55 ± 0.08**
Log(fat_fasting)				**0.75 ± 0.04**

*The heritabilities (±s.e.) are in diagonal and the genetic correlations (±s.e.) are above the diagonal. These genetic parameters were calculated using genomic data. The value in bold are considered as significant as their standard error is lower than half of the value*.

## Discussion

Isolating fish to estimate individual feed efficiency was primly done by Silverstein (2006) on 55 rainbow trout. The method we present is, however, the first to estimate individual FCR of a large number of fish (588) which allowed estimating genetic parameters of individual FCR. With this method based on individual estimation of feed intake and weight gain under restricted feeding in a 200 aquariums facility, we found phenotypic variability in FCR (FCR_aquarium), in daily growth coefficient (DGC_aquarium) and in daily feed intake coefficient (DFC_aquarium). The phenotypic coefficient of variation (CV) for FCR_aquarium (21%) was close to that observed by De Verdal et al. (2017) who estimated a CV of 23.4% for individual FCR in Nile tilapia with video observation over a period of 10 days. Silverstein (2006) also showed phenotypic variability of residual feed intake (RFI, a trait related to feed efficiency) in isolated rainbow trout. This is an encouraging result toward possible genetic improvement of FCR in sea bass, as our results also showed that FCR_aquarium, DGC_aquarium and DFC_aquarium were all heritable (genomic heritability estimates of 0.47, 0.76, and 0.57, respectively). Additionally, in these conditions of restricted feeding, FCR_aquarium and DGC_aquarium were strongly phenotypically and genetically correlated (*r*_p_ = −0.78 and *r*_g_ = −0.98). Such very high negative phenotypic correlation under restricted feeding was already observed by Silverstein (2006) in trout (*r*_p_ = −0.57 between RFI and growth). Moreover, such high negative genetic correlation was similar to the estimates obtained in pigs with −0.94 (Nguyen and McPhee, 2005) and in rabbits with −1.00 (Drouilhet et al., 2013) also evaluated in restricted feeding conditions. The heritability estimated for FCR using pedigree (0.25) was also similar to the heritability of FCR measured in the pig (0.16) and rabbit (0.23) studies. However, the heritability of growth rate was higher (0.39 with pedigree) compared to the pig study (0.16) and the rabbit study (0.22) but such high heritability for growth rate is common in fish and especially sea bass (e.g., 0.43 in Vandeputte et al., 2014).

In pigs and rabbits, the results were used to set up a selection procedure based on selection of faster growing animals under restricted feeding. They both showed that such selection resulted in an improvement of FCR in the next generations. In rabbits, FCR was reduced by 0.2 (from 2.82 to 2.63) after 9 generations (Drouilhet et al., 2016). In pigs, the EBV of FCR was reduced by 0.2 after 4 generations (Nguyen et al., 2005). Thus, selecting for higher growth rate under restricted feeding is efficient to reduce FCR in terrestrial livestock. Drouilhet et al. (2016) even showed that the correlated response obtained by this method was similar to the correlated response obtained from selection on residual feed intake under *ad libitum* feeding. This fact is also supported by similar estimates of heritabilities for FCR between the different feeding regimes, restricted and *ad libitum*, in pigs and rabbits (Hermesch, 2004; Drouilhet et al., 2013). Thus, the prospects to improve FCR by selection is also promising for sea bass using individual measurement of growth rate in isolation under restricted feeding. Nevertheless, rearing sea bass in isolation does not reflect commercial rearing conditions. To address this issue, we realized a validation experiment where we recorded the FCR of groups of fish in tanks. The tanks were composed of several fish previously phenotyped in individual aquariums. The effects of DGC_aquarium and DFC_aquarium on the group FCR were significant for two of the 3 weeks periods investigated and for the overall period of 9 weeks. These results support the hypothesis that DGC_aquarium under restricted feeding is a usable proxy of the FCR of fish in groups fed *ad libitum—*which is the standard rearing procedure. Silverstein (2006) also showed that individual and group performances were correlated in trout. It suggests therefore that selecting for better DGC_aquarium and lower DFC_aquarium would improve the FCR performance of fish in groups. However, a selection response experiment needs to be done to validate this point. The correlated response to selection obtained in FCR by Nguyen et al. (2005); Drouilhet et al. (2016) after selection on growth rate under restricted feeding was due to an increase of the body weight of animals in pigs. This increase in body weight was paired with a decrease of feed intake (Nguyen et al., 2005). They suggested that selection under restricted feeding would, therefore, increase the partitioning of energy for growth and decrease the partitioning of energy for maintenance. Cameron et al. (1994) also suggested that restricted feeding may select animals with higher partitioning of energy toward protein deposition rather than toward fat deposition. However, Kanis (1990) suggested that energy partition toward protein deposition was negatively associated with feed intake capacity. Hence, the selection procedure involving restricted feeding may not select for the animals with the maximal growth and then the maximal protein deposition rate. This could explain the moderate but not significant genetic correlation between DGC_aquarium and DGC_group of 0.13. The best overall selection objective may therefore require a little more emphasis on growth rate in group condition.

Our results on fat content, however, does not follow this hypothesis. Muscle fat content was indeed positively genetically correlated to DGC_aquarium. Additionally, muscle fat content was also positively correlated to DGC_fasting, which is a proxy of metabolic rate and feed efficiency as we showed that the most efficient fish in aquariums were coming from parents with lower weight loss during fasting (F– line) and that the tanks with the best FCR had lower averaged GEBV for DGC_fasting. This means that the fish that lost less weight during fasting and the fish that were more efficient in aquarium were genetically fatter. A potential explanation for these results is that the fish tolerant to fasting would express a more reactive behavior with lower swimming activity. Yet, we know that physical activity might be linked to energy required for maintenance as it has been observed in mice by Mousel et al. (2001) who found that selected mice for low heat loss (a measure of metabolic activity) had lower locomotor activity. This potential link between lower activity and tolerance to fasting could cause the higher fat content observed in our fish. This is supported by Simpkins et al. (2003) who showed that, during fasting, the fat content of rainbow trout was significantly higher for sedentary fish than for active fish. Hence, the fish losing less weight during fasting would, in fact, have lower metabolic activity causing their higher fat content and their better feed efficiency in aquarium and in groups.

These positive genetic correlations between fat content and weight loss at fasting follow earlier results of Grima et al. (2010b). These results are in contradiction with the commonly accepted theory that more efficient animals are leaner because protein deposition requires less energy than fat deposition per unit of wet weight gain (Knap and Kause, 2018). This theory is supported in fish by several studies on trout (Quillet et al., 2007; Kause et al., 2016), a species selected for several generations for higher growth rate. Yet, we know that selection for growth rate tends to generate more proactive animals (Sundström et al., 2004; Huntingford and Adams, 2005) that display a more aggressive behavior and higher exploratory capacities compared to wild or unselected animals for which bigger animals tend to be shy and reactive (Adriaenssens and Johnsson, 2010; Ferrari et al., 2016). In our study the sea bass were potentially more reactive than commercial populations selected for growth, as they were selected for 3 generations only (they had wild great grandparents) but only based on their weight loss during fasting and not on growth rate. Thus, from our results, DGC_aquarium, DGC_fasting, and muscle_fat could potentially be used in an index to select genetically superior animals for better feed efficiency. However, the direction to which muscle_fat should be improved remains uncertain and the relationship between muscle_fat, DGC_aquarium, and DGC_fasting needs to be verified on current commercially selected population.

Even though this new phenotyping method gave us essential information for the genetic improvement of FCR in sea bass, it is also a tedious and time consuming method. Over 6 months, we could only phenotype 588 fish. This small number of fish phenotyped caused a relatively low reliability of our genetic models. For instance, using 340 fish genotyped and phenotyped for DGC_aquarium to predict the performance of the 80 remaining fish (80–20% ratio), we reached a reliability of only 0.33 using ssGBLUP. This reliability was, however, much larger than the reliability realized with a PBLUP model using the same data (0.15). This indicates that the use of genomic data would be essential to enhance the prediction of EBV in selection candidates using a relatively small number of fish phenotyped for DGC_aquarium. Our reliability estimate with ssGBLUP was slightly lower than that of Bangera et al. (2017) for disease resistance in Atlantic salmon. In this study, they showed that the reliability of GEBV calculated with ssGBLUP for resistance to salmon rickettsial syndrome was about 0.41 when using 80% of the fish phenotyped and genotyped to predict the remaining 20%. Our results showed also that the reliability could be increased with more fish phenotyped as we did not reach a plateau when increasing the number of fish in the training group. However, this reliability results must be taken with care as the formula used to calculate the reliability is an approximation of the accuracy (Gunia et al., 2014). In order to estimate the true reliability of GEBV, the G3 fish phenotyped for DGC_aquarium and genotyped could be crossed to generate a G4 in a future experiment. Then, by phenotyping several fish of G4 for DGC_aquarium we could estimate a proxy of the true breeding value of G3 fish. Finally, the GEBV calculated previously could be correlated to these true breeding values to obtain a better estimate of the accuracy of ssGLUP model. Such procedure has been implemented in rainbow trout for resistance to bacterial cold water disease (Vallejo et al., 2017), and showed that the predictive ability of genomic predictions was twice higher than that of traditional pedigree BLUP. This confirms the importance of genomic data for genetic improvement of traits which are difficult to record, such as disease resistance and FCR.

An important aspect for the practical applicability of this method is therefore its cost-benefit ratio. Based on the present experiment, the cost of this selection method applied for 588 fish was about 50 k€: phenotyping costs 26 k€ and genotyping cost 24 k€ (60 € per fish on 400 G3 fish). While the cost of genotyping tends to decrease, the phenotyping cost remains important considering manpower (≈14 k€, 930 h at 15€/h over 6 months) and infrastructure costs (12 k€ per year, 200 aquariums and their recirculating system). The genetic gain for FCR obtained from this method has yet to be demonstrated with a response to selection experiment, but we can roughly estimate the response that could be achieved. The difference of group FCR between the best and the worst fish was about 2% (Figure 8), with an estimated heritability of 0.75 for DGC_aquarium, the potential gain per generation could be 1.5%. In an integrated fish farm (producing its own juveniles) that produces 3,000 tons of fish per year, feed consumption is about 4,500 tons of feed per year (FCR = 1.5) for a total cost of 6,750 k€ per year. It means that a gain of 1.5% would save the company about 100 k€/y, which is more than the cost of selection.

Despite the potential economic gain that could be achieved, we can point out that we could only phenotype juveniles of about 25 g to fit with the size of the aquariums (10 L). However, the targeted trait we wish to improve is FCR at commercial size because the animals consume higher amounts of feed in the later stages of production, hence further increasing the interest of improving FCR at such late stages. Based on our results, we cannot tell whether the most efficient fish when weighting 25 g will also be the most efficient at commercial size (450 g). Nevertheless, the measure of feed efficiency of groups took place when fish were 105 g till 200 g (on average) and the link between DGC_aquarium and FCR_group suggest that the most efficient fish early in life (in aquarium) tend to stay the most efficient later in life (in groups). Ideally, individual FCR should be measured a second time later in the fish life. However, previous experiments already revealed that bigger sea bass do not feed as easily as juveniles, or even do not feed at all when isolated (Ferrari et al., 2015). Therefore, such measure of growth rate in aquarium at commercial size is *a priori* not feasible in sea bass. An opportunity to overcome this issue would be to find traits that integrates the efficiency of the animals through its entire life. This could be done using mechanistic animal growth models. Such models aim at describing the growth of an individual based on underlying biological parameters to estimate energy uptake, storage, and utilization. These biological parameters of growth models can be, for instance, routine metabolic rate, or allocation to soma. Each of these biological parameters could, then, be generated for each individual by optimization of model's parameter. In our case, the optimization would be done by fitting the predicted weight and the predicted feed intake of an individual to its weight measured along its entire life and its feed intake measured as juveniles in aquarium. With this approach, we may be able to highlight potential genetic variation within a population and to find heritable model's parameters potentially related to the feed efficiency over an entire life. Such parameters could then be improved with a breeding program. A similar approach have been presented by Doeschl-Wilson et al. (2007) who used model inversion to obtain estimates of phenotypic and genetic components of the biological traits in a mechanistic growth animal model for pig. The results of this study suggest that such mechanistic growth models can be useful to animal breeding through the introduction of new biological traits that are less influenced by environmental factors than phenotypic traits currently used and that are valid all along the life of the individuals.

## Data Availability

The raw data supporting the conclusions of this manuscript can be found on SEANOE (https://doi.org/10.17882/58267).

## Ethics Statement

This study was carried out in accordance with the recommendations of Directive 2010-63-EU on the protection of animals used for scientific purposes. The protocoles were approved by C2EA−36 (Comité d'éthique en expérimentation animale Languedoc-Roussillon) under authorizations APAFIS#1362-2015071718471856_v4 and APAFIS#9877-2017042614262200_v2.

## Author Contributions

MB, FA, AV, BC, and MV designed the animal experiments. MB, AV, and FC performed the animal experiment. MB, FA, and MV performed the analysis. MB wrote the manuscript. MV and FA revised the manuscript. All authors read and approved the manuscript.

### Conflict of Interest Statement

The authors declare that the research was conducted in the absence of any commercial or financial relationships that could be construed as a potential conflict of interest.
