# Supplementary material for: Combining Individual Phenotypes of Feed Intake With Genomic Data to Improve Feed Efficiency in Sea Bass
Source: Front Genet. 2019 Mar 29;10:219. doi: 10.3389/fgene.2019.00219 (PMC6449465; doi:10.3389/fgene.2019.00219)
Supplement: Supplementary file 1 [file Table_1.pdf]

Supplementary Material

Combining Individual Phenotypes of Feed Intake With Genomic Data to Improve Feed Efficiency in Sea Bass

M. Besson[1,2,*], F. Allal[2], B. Chatain[2], A. Vergnet[2], F. Clota[1,2] & M. Vandeputte[1,2]

Supplementary Table 1. Chemical and nutritional compositions of the feed used during the measurement of individual feed conversion ratio in aquarium.

Chemical composition	
Proteins	47 %
Lipids	18 %
Moist	8 %
Cellulose	1.4 %
Phosphorus	1.1 %
Ash	8.5 %
Nitrogen-free extract	17.1 %
Nutritional composition	
Crude energy	4990 kcal (21 MJ) per kg of feed
Digestible energy	4541 kcal (19 MJ) per kg of feed
Ratio digestible protein / digestible energy	30 $g.MJ^{-1}$ per kg of feed
