# Supplementary material for: Combining Individual Phenotypes of Feed Intake With Genomic Data to Improve Feed Efficiency in Sea Bass
Source: Front Genet. 2019 Mar 29;10:219. doi: 10.3389/fgene.2019.00219 (PMC6449465; doi:10.3389/fgene.2019.00219)
Supplement: Supplementary file 2 [file Table_2.pdf]

Supplementary Material

Combining Individual Phenotypes of Feed Intake With Genomic Data to Improve Feed Efficiency in Sea Bass

M. Besson[1,2,*], F. Allal[2], B. Chatain[2], A. Vergnet[2], F. Clota[1,2] & M. Vandeputte[1,2]

Supplementary Table 2. Analysis of covariance of average weight loss at fasting in G3 (DGC_fasting).

	Df	Type "III" Sum Sq	F value	p value
fat_pre_fasting	1	0.066	6.10	0.013
Sire origin	1	0.009	0.85	0.35
Dam origin	1	0.144	13.38	0.0002
fat_pre_fasting:sire origin	1	0.0004	0.03	0.85
fat_pre_fasting:dam origin	1	0.065	6.02	0.014
Sire origin:dam origin	1	0.001	0.12	0.72
fat_pre_fasting:sire origin:dam origin	1	0.0004	0.04	0.83
Residuals	693			
