# Supplementary material for: Combining Individual Phenotypes of Feed Intake With Genomic Data to Improve Feed Efficiency in Sea Bass
Source: Front Genet. 2019 Mar 29;10:219. doi: 10.3389/fgene.2019.00219 (PMC6449465; doi:10.3389/fgene.2019.00219)
Supplement: Supplementary file 3 [file Table_3.pdf]

Supplementary Material

Combining Individual Phenotypes of Feed Intake With Genomic Data to Improve Feed Efficiency in Sea Bass

M. Besson[1,2,*], F. Allal[2], B. Chatain[2], A. Vergnet[2], F. Clota[1,2] & M. Vandeputte[1,2]

Supplementary Table 3. Least square mean (±s.e) of the GEBV of DGC_fasting as function of parental origins within generation 2 and generation 3.

GEBV of DGC_fasting	Parental origin				Significance level
	F-/F-	F-/F+	F+/F-	F+/F+	Parental_origin
Generation 2	0.033 (0.0013)	-	-	-0.006 (0.0012)	< 0.0001
Generation 3	0.059 (0.002)	-0.0024 (0.0019)	0.013 (0.002)	-0.041 (0.0019)	< 0.0001
