# Supplementary material for: Combining Individual Phenotypes of Feed Intake With Genomic Data to Improve Feed Efficiency in Sea Bass
Source: Front Genet. 2019 Mar 29;10:219. doi: 10.3389/fgene.2019.00219 (PMC6449465; doi:10.3389/fgene.2019.00219)
Supplement: Supplementary file 4 [file Table_4.pdf]

Supplementary Material

Combining Individual Phenotypes of Feed Intake With Genomic Data to Improve Feed Efficiency in Sea Bass

M. Besson[1,2,*], F. Allal[2], B. Chatain[2], A. Vergnet[2], F. Clota[1,2] & M. Vandeputte[1,2]

Supplementary Table 4. Bivariate regression analysis of FCR of tanks (FCR_group) on the average phenotypic value of the fish in each tank for daily growth coefficient in aquarium (avg_DGC_aquarium) and daily feed intake coefficient (avg_DFC_aquarium), for each of the three periods of three weeks during the validation experiment and the full period of nine weeks. In italic is the regression coefficient (± s.e.).

Effects	Significance level (type II p-value)			
	Group_P1	Group_P2	Group_P3	Group_full
log(avg_DGC_aquarium)	*-0.19 ±0.05*<br>$F_{1,9}$ = 16.58 p = 0.003	*-0.17 ±0.02*<br>$F_{1,9}$ = 8.29 p = 0.018	*-0.007 ±0.05*<br>$F_{1,9}$ = 0.02, p = 0.89	*-0.12 ±0.009*<br>$F_{1,9}$ = 11.60, p = 0.008
log(avg_DFC_aquarium)	*0.4 ±0.09*<br>$F_{1,9}$ = 18.81, p = 0.002	*0.30 ±0.06*<br>$F_{1,9}$ = 6.97, p = 0.027	*-0.03 ±0.1*<br>$F_{1,9}$ = 0.08, p = 0.77	*0.22 ±0.07*<br>$F_{1,9}$ = 10.13, p = 0.011
