# Supplementary material for: Combining Individual Phenotypes of Feed Intake With Genomic Data to Improve Feed Efficiency in Sea Bass
Source: Front Genet. 2019 Mar 29;10:219. doi: 10.3389/fgene.2019.00219 (PMC6449465; doi:10.3389/fgene.2019.00219)
Supplement: Supplementary file 5 [file Table_5.pdf]

Supplementary Material

Combining Individual Phenotypes of Feed Intake With Genomic Data to Improve Feed Efficiency in Sea Bass

M. Besson[1,2,*], F. Allal[2], B. Chatain[2], A. Vergnet[2], F. Clota[1,2] & M. Vandeputte[1,2]

Supplementary Table 5. Bivariate regression analysis of FCR of tanks (FCR_group) on the average genomic estimated breeding value of the fish in each tank for daily growth coefficient during fasting (avg_GEBV_DGC_fasting) for each of the three periods of three weeks during the validation experiment and the full period of nine weeks. In italic is the regression coefficient (± s.e.).

Effects	Significance level			
	Group_P1	Group_P2	Group_P3	Group_full
avg_GEBV_DGC_fasting	*-2.84 ±0.78*<br>$F_{1,14}$ = 13.17, p = 0.003	*-1.70 ±0.61*<br>$F_{1,14}$ = 7.68, p = 0.015	*-0.58 ±0.75*<br>$F_{1,12}$ = 0.60, p = 0.45	*-1.73 ±0.58*<br>$F_{1,12}$ = 8.74, p = 0.012
